# Crescentic Glomerulonephritis: Pathogenesis and Therapeutic Potential of Human Amniotic Stem Cells

**DOI:** 10.3389/fphys.2021.724186

**Published:** 2021-10-15

**Authors:** Ahmed Al Mushafi, Joshua D. Ooi, Dragana Odobasic

**Affiliations:** Department of Medicine, Monash Medical Centre, Centre for Inflammatory Diseases, Monash University, Clayton, VIC, Australia

**Keywords:** chronic kidney disease, crescentic glomerulonephritis, human amniotic stem cells, inflammation, immunity

## Abstract

Chronic kidney disease (CKD) leads to significant morbidity and mortality worldwide. Glomerulonephritis (GN) is the second leading cause of CKD resulting in end stage renal failure. The most severe and rapidly progressive type of GN is characterized by glomerular crescent formation. The current therapies for crescentic GN, which consist of broad immunosuppressive drugs, are partially effective, non-specific, toxic and cause many serious side effects including infections, cancer, and cardiovascular problems. Therefore, new and safer therapies are needed. Human amniotic epithelial cells (hAECs) are a type of stem cell which are isolated from the placenta after birth. They represent an attractive and novel therapeutic option for the treatment of various inflammatory conditions owing to their unique and selective immunosuppressive ability, as well as their excellent safety profile and clinical applicability. In this review, we will discuss the immunopathogenesis of crescentic GN, issues with currently available treatments and how hAECs offer potential to become a new and harmless treatment option for this condition.

## Introduction

The immune system and the kidneys are closely linked, and chronic kidney disease (CKD) often results from various auto(immune) disorders. CKD is due to slowly progressive, chronic deterioration of kidney function. Crescentic glomerulonephritis (GN) is a chronic immune-mediated disease which causes severe glomerular inflammation and injury, and often leads to irreversible kidney failure. It is a common cause of morbidity and mortality worldwide. GN is a major contributor to the escalating health burden associated with CKD.

Almost all current therapeutic concepts in autoimmune diseases are based on the systemic suppression of immune functions and are not curative. Currently used immunosuppressive therapies for crescentic GN are only partially effective, toxic and provide broad, non-specific immunosuppression, thus producing significant adverse effects. These including mainly severe infections, but also cancer and cardiovascular events. It is these treatment-caused side effects which cause the majority of patient deaths. Therefore, new and safer therapies are much needed.

Human amniotic epithelial cells (hAECs) are a type of stem cell which are isolated from the human placenta after birth. They are an attractive and novel therapeutic option for the treatment of crescentic GN due to their (i) ethical, non-invasive, and speedy isolation from the amniotic membrane of the placenta which results in an abundance of readily available cells, (ii) potent immunosuppressive capacity, (iii) low immunogenicity (ability to activate the immune system) and (iv) their ability to fight microbes and cancer and protect against cardiovascular disease. hAECs have attenuated various inflammatory diseases in mouse models, without being rejected, producing tumors or causing any major side effects. They are currently being tested in several clinical trials to treat different conditions including lung and liver disease and stroke. The purpose of this review is to discuss and link some of the major immune and inflammatory mechanisms to the progression of crescentic GN, discuss the common therapies and their limitations, and outline the therapeutic potential of hAECs in safely reducing glomerular injury.

## Crescentic Glomerulonephritis

The term glomerulonephritis (GN) refers to immune-mediated inflammation of the renal glomeruli. GN is diagnosed based on clinical presentation, etiology, histopathology or pathogenesis. Most patients present with hematuria, proteinuria, and impaired glomerular filtration rate (Chadban and Atkins, 2005). This pathological condition can be acute or chronic (developing over several months to years) based on the timing of clinical presentation (Mejia-Vilet and Parikh, 2019). Acute GN that develops into rapidly progressive disease most often results from conditions that involve an abnormal immune reaction. Sometimes, acute GN does not resolve, and instead becomes long lasting (chronic) (Vinen and Oliveira, 2003).

Crescentic GN is a severe form of glomerulonephritis characterized by the destruction of the renal glomeruli that often lead to end-stage renal disease over a relatively short period of time (days, weeks, or months) (Jennette and Thomas, 2001; Jennette, 2003). It is characterized morphologically by extensive crescent formation, defined as two or more cell layers in Bowman’s or urinary space (Jennette and Thomas, 2001; Parmar and Bashir, 2019). Crescents are formed by infiltrating and proliferating immune and local cells, along with deposited fibrin. This occurs after the disruption of the glomerular structure, which allow for circulating cells, inflammatory cytokines, and blood proteins to pass through the blood vessel wall into the Bowman space. The major components in the glomerular crescent are procoagulant factors, macrophages, T cells, fibroblasts, and parietal and visceral epithelial cells (Karras, 2018; Tsui et al., 2018).

On the basis of immunopathological findings, crescentic GN can be classified into three major categories: anti-glomerular basement membrane (GBM) antibody disease (Goodpasture’s syndrome), immune complex GN (e.g., lupus nephritis), and pauci-immune GN which is often associated with anti-neutrophil cytoplasmic autoantibodies (ANCA) and is thus also known as ANCA-associated vasculitis (AAV) (Jennette and Thomas, 2001; Parmar and Bashir, 2019). Anti-GBM autoantibodies are highly specific for Goodpasture’s disease, in which they are generally directed against the non-collagenous (NC1) domain of the alpha 3 chain of type IV collagen [α3(IV)NC1] (Gulati and McAdoo, 2018). These immunoglobulins cause glomerular capillary wall damage by local complement activation and neutrophils. T-cells also play a distinct pathogenic role in driving cell-mediated destruction of the glomeruli in this disease (Dean et al., 2005; McAdoo and Pusey, 2017). Environmental factors, like smoking, hydrocarbons and exposure to high oxygen, are thought to increase the likelihood of developing anti-GBM (McAdoo and Pusey, 2017. Anti-GBM GN accounts for around 10–15% of all cases of crescentic GN (McAdoo and Pusey, 2017). Immune complex GN, which comprises 25–30% of all cases crescentic GN, is characterized by a granular pattern of immune complex deposition in glomeruli (Naik and Shawar, 2020). AAV is associated with ANCA specific for neutrophil proteins, predominantly myeloperoxidase (MPO) or proteinase 3 (PR3) (Dey et al., 2016). This is the most common form of crescentic GN, contributing to about 65-70% of all cases (Naik and Shawar, 2020), and it refers to a necrotizing/crescentic GN with few or no immune deposits in glomeruli, as detected by immunofluorescence (Syed et al., 2015).

### Pre-clinical Models of Crescentic Glomerulonephritis

Experimental evidence for the pathogenesis of crescentic GN comes mainly from pre-clinical models, including lupus nephritis, experimental autoimmune GN (EAG; model of Goodpasture’s disease), MPO-ANCA vasculitis and nephrotoxic nephritis (NTN).

#### Nephrotoxic Nephritis

A large body of evidence about the immunopathogenesis of crescentic GN has come from one of the most widely used and best-characterized models, called nephrotoxic nephritis (NTN), also known as autologous anti-GBM globulin GN. In this model, rodents such as rats, mice and rabbits are passively injected with foreign polyclonal antibodies (globulins) targeting the mouse GBM (Odobasic et al., 2014). It comprises of two distinct phases. The first phase is called the heterologous phase and is associated with transient glomerular injury and inflammation due to the binding of injected foreign antibodies in a linear fashion to the GBM, before the development of an adaptive immune response (Odobasic et al., 2014). This phase is characterized by a neutrophil influx which peaks around 2 h after the injection of transferred antibodies, and proteinuria, peaking within the first 24 h (Schrijver et al., 1990; Tipping et al., 1994; Odobasic et al., 2014). Neutrophils contribute to glomerular damage by producing several inflammatory mediators including reactive oxygen species (ROS), proteases (Johnson et al., 1988; Couser, 1998), and MPO (Odobasic et al., 2007). Accumulation of neutrophils in inflamed glomeruli is dependent on intraglomerular expression of adhesion molecules, P-selectin, and intercellular adhesion molecule-1 (ICAM-1) (Tipping et al., 1994; Nomura et al., 1996; Saleem et al., 1998). The pathogenic role of neutrophils in glomerulonephritis is based on the production of several inflammatory mediators including ROS and protease such as elastase, proteinases and cathepsin G (Johnson et al., 1988; Couser, 1998).

The second phase is called the autologous phase and tends to begin about 6 to 7 days later. It is caused by an adaptive immune response to the foreign anti-GBM globulin (Odobasic et al., 2014; Ougaard et al., 2018). This phase is characterized by more severe injury and crescent formation due to the presence of cellular effectors including CD4 + T cells, macrophages, neutrophils and fibrin (Huang X.-R. et al., 1997; Huang X. et al., 1997).

In mice, an accelerated model of NTN can be induced by pre-immunizing mice with normal sheep globulin (NSG) (Ougaard et al., 2018). In accelerated NTN, the use of adjuvants which contain dead mycobacteria are included in the pre-immunization to improve the immune response (Ougaard et al., 2018). This pre-immunization produces a strong T and B cell response to sheep globulin and gives rise to an immediate autologous phase once sheep anti-GBM antibodies are administered (Odobasic et al., 2014). For animal models without pre-immunization, injury is less acute compared to the pre-immunized accelerated model and termed as a non-accelerated model (Odobasic et al., 2014).

#### Experimental Models of MPO-AAV, Lupus Nephritis, and Goodpasture’s

Other models of crescentic GN exist and they have been also widely used to reveal pathogenetic mechanisms of disease. MPO-AAV can be induced in mice by immunizing animals with MPO which results in the development of active anti-MPO autoimmunity, followed by neutrophil lodgment in glomeruli and deposition of the autoantigen for subsequent recognition by MPO-specific T cells (Ruth et al., 2006; Odobasic et al., 2019). Alternatively, glomerular injury can be initiated by passive transfer of anti-MPO antibodies (Xiao et al., 2002; Ooi et al., 2014). Experimental autoimmune GN (EAG), a model of Goodpasture’s disease, can be induced in mice by repeated immunization with α3(IV)NC1 (Ooi et al., 2009). Several models of lupus nephritis exist in which susceptible mice such as MRL/lpr and NZB/NZWF1 spontaneously develop disease (Richard and Gilkeson, 2018). Although none of these models fully recapitulate human disease, they all (including NTN) closely resemble crescentic GN seen in patients, both immunologically and pathologically, and are therefore invaluable pre-clinical tools to study disease pathogenesis and test new potential therapies.

### Mechanisms of Pathogenesis of Crescentic Glomerulonephritis

**FIGURE 1:**
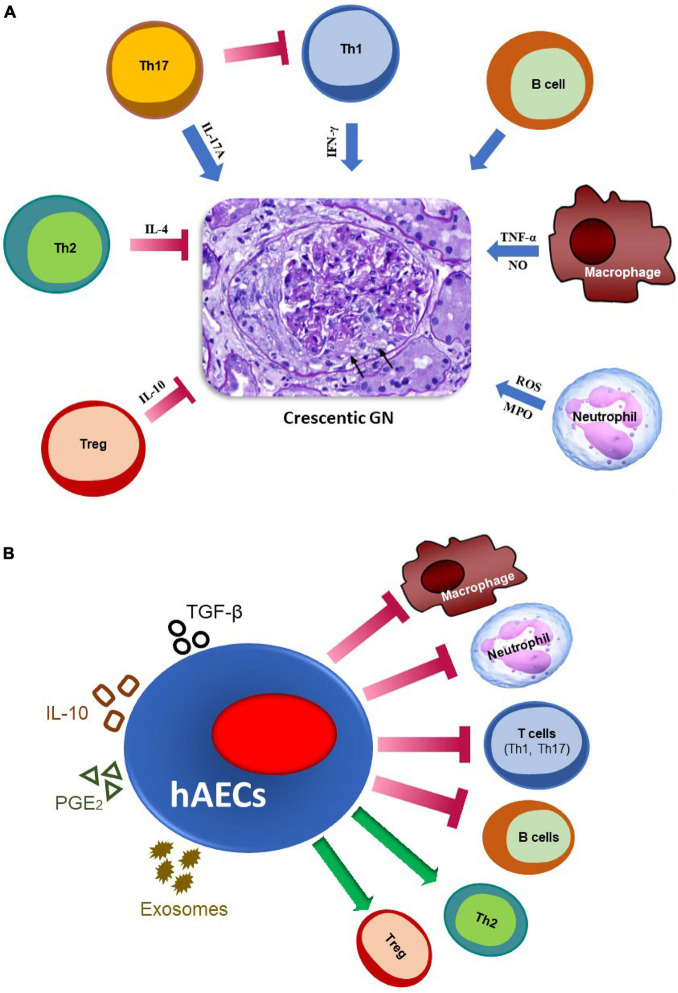
The major immune pathways involved in the pathogenesis of crescentic GN and mechanisms by which hAECs may attenuate glomerular injury. **(A)** Effector immune mechanisms in crescentic GN. Macrophages contribute by secreting nitric oxide (NO) and TNF-α, while neutrophils contribute by producing reactive oxygen species (ROS) and releasing myeloperoxidase (MPO) enzyme. IFN-γ produced by Th1 cells mediates crescent formation. IL-17A (a Th17 cytokine) promotes early stage of kidney injury, but attenuates established disease by inhibiting Th1 responses. B cells, which act as antigen-presenting cells and produce autoantibodies, also promote glomerular injury. On the contrary, IL-4 producing Th2 and IL-10-producing regulatory T cells (Tregs) play a protective role in this disease. Arrows indicate glomerular crescent formation. **(B)** Potential mechanisms by which hAECs may reduce inflammation and damage in glomeruli. hAECs suppress activation and/or infiltration of pro-inflammatory T cells (Th1 and Th17), B cells, macrophages and neutrophils, while promoting inhibitory cells such as Tregs and Th2. They exert their effects by producing anti-inflammatory mediators such as TGF-β, IL-10 and PGE2, and releasing exosomes, to restrict kidney injury.

Multiple immune mechanisms contribute to the pathogenesis of crescentic GN. A summary of the main immune pathways which positively or negatively regulate the development of crescentic GN is shown in Figure 1A.

#### The Role of Innate Immunity – Neutrophils and Macrophages

Innate and adaptive immune system activation are a common underlying mechanism for several forms of crescentic GN (Imig and Ryan, 2013). Studies in the NTN model using neutrophil depletion have shown that these cells contribute to glomerular injury during the early autologous phase of disease (Disteldorf et al., 2015). Macrophages are the dominant effector cells present in the kidney which mediates glomerular crescent formation and kidney damage in all models of crescentic GN (Lan et al., 1997; Odobasic et al., 2014; Rousselle et al., 2017). Their accumulation within glomeruli has been blocked by administration of polyclonal sheep anti-rabbit macrophage serum in models of anti-GBM disease, leading to prevention of the development of GN (Holdsworth et al., 1981). Moreover, a micro-encapsulated approach in which macrophages are depleted by clodronate has resulted in reducing renal damage in anti-GBM GN (D’Souza et al., 1999). Further studies have shown that macrophages can induce glomerular injury by several mechanisms including deposition of fibrin (which impairs glomerular filtration) and production of various proinflammatory mediators including ROS, reactive nitrogen species such as nitric oxide (NO) and cytokines such as IL-1, TNF-α, and macrophage migration inhibitory factor (MIF) (Imig and Ryan, 2013).

#### The Role of Adaptive Immunity – T Cells and B Cells

Numerous studies in various models of crescentic GN have shown that CD4 + T cells play a critical role in this disease (Tipping et al., 1998; Dean et al., 2005; Ruth et al., 2006). CD4 T helper cells (Th) can be divided into several subsets, with the major and best-understood ones being Th1, Th17 and Th2, mainly characterized by the expression of IFNγ, IL-17A, and IL-4, respectively. Several studies have indicated that crescentic anti-GBM globulin GN is driven by Th1 cells, while Th2 cells reduce the severity of disease. For instance, GN induced in mice with a predominant Th1 response (C57BL/6) shows severe crescentic formation with prominent glomerular T cell and macrophage infiltration and fibrin deposition which are associated with elevated IFN-γ and weak IL-4 production (Huang X.-R. et al., 1997). On the other hand, in Th2-prone (BALB/c) mice, crescent formation as well as glomerular T cell and macrophage influx were relatively low (Huang X.-R. et al., 1997). In addition, mice lacking endogenous IFN-γ developed less severe GN than genetically normal C57BL/6 mice, whereas IL-4-deficient mice developed more severe crescentic GN associated with increased accumulation of T cells and macrophages in glomeruli (Kitching et al., 1998, 1999). While IFNγ is protective in EAG (Kitching et al., 2004), the Th1 pathway has also been shown to promote glomerular crescent formation and kidney injury in experimental lupus nephritis and MPO-AAV (Richards et al., 2001; Summers et al., 2011).

Moreover, it has become clear that the Th17 pathway is important in the development of crescentic GN. In experimental MPO-AAV, mice lacking IL-17A are protected from early glomerular injury (Gan et al., 2010). Similarly, several lines of evidence, coming from using IL-17A-deficient mice or infusion of Th17 cells, have shown that the Th17 pathway promotes early glomerular injury in NTN (Paust et al., 2009; Summers et al., 2009; Odobasic et al., 2011), in line with results from experimental autoimmune uveitis (Luger et al., 2008). On the contrary, the Th1 pathway acts later in the disease process and produces more severe GN (Odobasic et al., 2011). Interestingly, local IL-17A production is protective in crescentic GN (Hamour et al., 2015), and in later stages of the disease (which is mediated by Th1), IL-17A can attenuate injury by systemically inhibiting Th1 responses (Odobasic et al., 2011).

Tregs are a specialized inhibitory subset of CD4 + T cells characterized by expression of CD25 and Foxp3. They play a key role as negative regulators of pathogenic immunity in crescentic GN. Studies using Foxp3-GFP reporter mice explored the functional role of T-regulatory cell in inhibiting anti-GBM nephritis (Ooi et al., 2011). Foxp3 protected against renal damage in anti-GBM GN induced in antigen-primed Foxp3-transgenic mice, via boost of Treg numbers and activity, and inhibition of Th immune responses at the systemic level and at sites of tissue injury (Yang et al., 2017). Similarly, Tregs are protective in other models of crescentic GN including MPO-AAV and lupus nephritis (Humrich et al., 2010; Tan et al., 2013; Odobasic et al., 2019). Further studies have shown that Tregs attenuate crescentic GN by releasing a potent anti-inflammatory cytokine, IL-10 (Ostmann et al., 2013).

In addition to T cells, B cells and autoantibodies have also been demonstrated to play a major role in the development of crescentic GN. B cells and/or antibodies against various autoantigens including MPO in AAV, α3(IV)NC1 in Goodpasture’s and nuclear antigens in lupus, are pathogenic in experimental crescentic GN (Xiao et al., 2002; Dean et al., 2005; Richard and Gilkeson, 2018). In contrast, evidence coming from μ-chain knockout mice, which lack mature B cells and cannot produce immunoglobulin, has shown that glomerular antibody deposition is not essential for crescent formation in response to the planted glomerular antigen in the NTN model (Li et al., 1997). B cells are well known to promote immune-mediated injury by several mechanisms including acting as antigen-presenting cells to activate T cells and differentiating into autoantibody-producing plasma cells.

#### Pathogenic Mechanisms in Human Crescentic Glomerulonephritis

In human studies, strong evidence from patients’ kidney biopises, and blood samples has accumulated to support the role of immune cells and antibody in cresentic GN, with findings similar to those in animal models. While neutrophils are prominent in cresentic GN (Kaplan, 2013), a considerable body of evidence has emerged to show that CD4 + T cells and macrophages are critically involved in all patterns of human cresentic glomerulonephritis (Stachura et al., 1984; Bolton et al., 1987; Nolasco et al., 1987; Müller et al., 1988; Cunningham et al., 1999). Although many limitations have constrained the assessment of nephritogenic responses in human GN, a number of studies have indicated that cresentic glomerulonephritis is a manifestation of a Th1 predominant delayed type hypersensitivity (DTH)-mediated immune response associated with predominant infiltration of macrophages and deposition of fibrin (Kitching et al., 2000). Furthermore, patients with crescentic GN have increased serum levels of IL-17A in comparison to healthy individuals, suggesting a role for the Th17 pathway in disease pathogenesis (Lu et al., 2017; Herrnstadt and Steinmetz, 2020). Human studies in AAV, SLE, and Goodpasture’s syndrome have revealed that dysregulation of Treg homeostasis and function is also associated with the development of crescentic GN (Herrnstadt and Steinmetz, 2020). Finally, B cells and autoantibodies against various endogenous targets in crescentic GN are pathogenic in human disease (Falk and Jennette, 1988; McAdoo and Pusey, 2017).

### Current Therapies for Crescentic Glomerulonephritis

In general, crescentic GN therapy is often classified into two phases. First is the activation of remission phase during the acute period, followed by the subsequent phase of maintenance therapy to control the underlying long-term immunopathology (Parmar and Bashir, 2017). The induction treatment is mainly composed of high-dose corticosteroids and intravenous (i.v.) pulse cyclophosphamide with the purpose of blocking the active inflammation and reducing the cellular and humoral immune response (Jennette, 2003; Moroni and Ponticelli, 2014). In addition, therapeutic plasma apheresis (plasma exchange) is a form of treatment that is frequently used to treat almost all types of crescentic GN by removing pathogenic autoantibodies (Flossmann et al., 2011; Kallenberg, 2014; Prendecki and Pusey, 2019).

Corticosteroids, synthetic drugs which closely resemble cortisol hormone, have been used for decades to modulate inflammation therapeutically by decreasing the movement of neutrophils to the inflammatory sites and inducing a transient lymphocytopenia (Olnes et al., 2016). This medication inhibits the expression and action of most proinflammatory cytokines, adhesion molecules, and suppresses MHC expression (Ponticelli and Locatelli, 2018). Cyclophosphamide, a cytostatic drug, is one of the oldest anti-cancer drugs and is widely used in therapy of crescentic GN by reducing the activity of the immune system (Jhaveri et al., 2013). Cyclophosphamide therapy significantly reduces total neutrophil, macrophage and lymphocyte counts (Jhaveri et al., 2013; Ménétrier-Caux et al., 2019).

Combination therapy with pulse cyclophosphamide plus pulse methyl prednisolone therapy significantly reduce proteinuria, serum creatinine, the level cellular crescents in crescentic GN (Tumlin et al., 2003), and present a greater reduction in the risk for ESRD.

However, induction therapy (corticosteroids with cyclophosphamide) is only partially effective, non-specific and produces many toxicities and serious side effects. Therefore, there is a major unmet need for safer, effective therapies. For example, in AAV, this treatment induces remission in 70–90% of patients, but the incidence of dialysis or death at 5 years is still high (∼30%), with the majority of early deaths caused by drug-related side effects, mainly infections caused by broad immunosuppression (Flossmann et al., 2011; Kallenberg, 2014; King and Harper, 2017). In lupus nephritis, remission is achieved in 50–60% of patients at best (Menez et al., 2018), relapse occurs in up to 25% of cases (Menez et al., 2018; Anders et al., 2020) and despite optimal care, many patients (up to 20%) develop kidney failure which requires renal transplantation or dialysis for survival (Menez et al., 2018; Anders et al., 2020). The unwanted adverse effects caused by these drugs include predominantly severe infections, as well as increased risk of malignancy, bone disease, dysglycemia, obesity, hypertension, mental problems, gastrointestinal bleeding, cataracts, and long-term risks of developing cardiovascular disease (King and Harper, 2017; Jefferson, 2018). More recently, rituximab, a pan B cell depleting anti-CD20 monoclonal antibody, has been approved for use instead of cyclophosphamide as a second line of treatment, particularly in AAV. However, although it is not inferior to cyclophosphamide in reducing disease, it induces similar rates of adverse effects, mainly infections, most likely due to hypogammaglobulinaemia and late onset neutropenia (Jones et al., 2015; Santos et al., 2020).

Adalimumab, an anti-TNFα monoclonal antibody, has been used as an adjunct therapy to standard immunosuppression. It can be an effective therapy for the induction of remission in AAV and may permit reduced prednisolone dosing, thus decreasing therapy-related toxicity (Laurino et al., 2010). However, other anti-TNFα agents, infliximab and etanercept, failed to demonstrate a benefit for remission maintenance in patients with AAV (Booth et al., 2004; Wegener’s Granulomatosis Etanercept Trial (WGET) Research Group, 2005).

Mycophenolate mofetil (MMF), is a salt form of the immunosuppressive drug mycophenolic acid. It inhibits the releasing of proinflammatory cytokines, nitric oxide, and LDH in macrophages and suppresses proliferation and infiltration of both T and B lymphocytes (Allison and Eugui, 2000). Recent studies, indicate that MMF may be as or even more effective in reducing proteinuria and hematuria and less toxic compared with cyclophosphamide in LN (Hu et al., 2002).

## Stem Cell-Based Therapy for Autoimmune and Inflammatory Diseases

Stem cell-based therapy is an attractive approach to ameliorate a broad range of human diseases and injuries, and it has been proven to be safe and effective in a wide range of immune-mediated diseases (Jin et al., 2014). Several types of stem cells, including mesenchymal, induced pluripotent and embryonic (Ryu et al., 2020), have reduced organ injury in models of inmune diseases and their potential side effects and efficiency have been assessed in clinical trials (Mûzes and Sipos, 2019).

### Mesenchymal, Embryonic, and Induced Pluripotent Stem Cells

Mesenchymal stem cells (MSCs) are multipotent adult stem cells that exist in various locations including umbilical cord, bone marrow and adipose tissue (Murray and Péault, 2015). MSCs have been successfully applied in treating a vast array of inflammatory and autoimmune conditions such as graft-versus-host disease (GVHD), multiple sclerosis (MS), type 1 diabetes (T1D), inflammatory bowel diseases (IBD), systemic lupus erythematosus (SLE), rheumatoid arthritis (RA), lung fibrosis, liver, and pancreatic fibrosis (Ryu et al., 2020). These cells modulate the immune reaction directly through regulating various immune cells including dendritic cells (DCs), macrophages, B cells, T cells and neutrophils, and by producing suppressive cytokines such as IL-10, TGF-β, and IL-35, as well as expresssing inhibitory ligands and receptors (e.g., PD-L1 and PD-1) (Jiang and Xu, 2020). MSCs can attenuate the development of crescentic GN (Thakkar et al., 2017) by decreasing neutrophil and macrophage recruitment to the kidney and promoting the phenotypic switching of renal macrophages to immunoregulatory cells (Furuhashi et al., 2013). In addition, the beneficial effects of MSCs in GN appear to be mediated by modification of the Th1/Th2 and/or Th17/Treg balance and inhibition of B-cell activation, as well as stimulation of IL-10, IL-4, foxp3, prostaglandin E2 (PGE2), and TGF-β production (Ma et al., 2013; Suzuki et al., 2013). Hence, MSCs have demonstrated capacity to attenuate experimental crescentic GN, but they also have a few disadvantages and carry some risks. For example, some methods of MSC isolation are invasive (e.g., obtaining MSCs from the bone marrow or adipose tissue). MSCs also require long-term culture (weeks) to expand adequate cell numbers for infusion. This increases the cost and risk of *in vitro* mal-transformation. It is also not optimal for the treatment of diseases such as crescentic GN in which early treatment is required to stop the progression of rapidly progressing deterioration of kidney function.

Embryonic and induced pluripotent stem cells (ESCs and iPSCs) are pluripotent stem cells. ESCs are isolated from the inner cell mass of blastocysts, while iPSCs are produced from adult somatic cells that are genetically reprogrammed to an ESC-like state by transcription factors (Ryu et al., 2020). It has been reported that the ESC-loaded gelatin microcryogels on rats slowed down the progression of CKD and alleviated glomerular injury (Geng et al., 2016). Likewise, administration of iPSCs in models of CKD preserved residual renal function by decreasing macrophage infiltration, upregulating TGF-β, inhibiting apoptosis and regulating cell proliferation and death signaling (Caldas et al., 2017; Sheu et al., 2020). Thus, ESCs and iPSCs may be able to reduce glomerular damage and retard the progression of CKD, but they also have some disadvantages. For instance, isolation of ESCs poses obvious ethical concerns, while iPSCs carry the risk of tumor development (Caldas et al., 2017).

### Human Amniotic Epithelial Cells

One particular stem cell type, hAECs, have attracted much attention in the recent years as an ideal therapuetic option for the treatment of autoimmune and inflammatory disorders due to their immunosuppresive ability and their superior safety and clinical applicability over other stem cell types. hAECs are a heterogeneous epithelial population that originates from the lining of the inner membrane of the placenta which provides an abundant cellular source for stem cell-based therapy (Miki et al., 2005). In comparison to other sources of stem cells, gestational tissue gives great advantages including easy collection without the need for invasive methods (Qiu et al., 2020). Large numbers of hAECs can be isolated from the placenta after birth, thus bypassing ethical barriers and resulting in an abundance of immediately available, primary (non-cultured) cells to be used therapeutically. These epithelial stem cells are pluripotent and have the capability of self-renewal and differentiating into all three germ layers, including the ectoderm, mesoderm, and endoderm (Miki et al., 2005). hAECs have immense potential to safely reduce the burden of many serious diseases and injuries to different organs, including the kidney, due to their unique properties (Ren et al., 2020). They possess some degree of plasticity, immune privilege, non-tumorigenicity, anti-infection/cancer properties, and lack of ethical concerns and paracrine properties that are essential to their potential therapeutic applications in immune-mediated diseases (Qiu et al., 2020).

#### Low Immunogenic Profile

Human Leukocyte antigens (HLA), encoded by the major histocompatibility complex (MHC) gene complex in humans, are the major molecules that initiate graft rejection (Mahdi, 2013). However, low HLA class-I (HLA-A, HLA-B, and HLA-C) expression and HLA class-II (HLA-DR) on hAEC surface have been identified, resulting in a low immunogenic profile upon transplantation (Hori et al., 2006). hAECs express non-classical HLA-G, which is thought to protect the fetal semi-allograft from maternal immune system rejection (Lefebvre et al., 2000). Expression of HLA-G confers a degree of immune privilege by suppressing natural killer cells, inducing apoptosis of activated CD8 + T cells and inhibiting CD4 + T cell proliferation (Banas et al., 2008).

#### Low Risk of Tumor Formation

Tumorigenicity is a common obstacle for cell-based therapies, since some cells may cause formation of tumors due to being immortal. Unlike ESCs and iPSCs (Ben-David and Benvenisty, 2011), hAECs do not express telomerase reverse transcriptase (Miki et al., 2005), which is a catalytic subunit of the telomerase enzyme playing a central role in tumorigenesis (Daniel et al., 2012). Therefore, hAECs do not promote tumor formation after transfer into recipients.

#### Immunosuppressive Capacity

Consistent with the role of the placenta to protect the fetus from being attacked by the maternal immune system during pregnancy, hAECs are immunosupressive. Similar to MSCs, the beneficial effects of hAECs are primarily mediated via their paracrine actions and not by their differentiation into target cells (Tögel et al., 2005; Wang et al., 2014). hAECs have blocked the immue system by suppressing effector T cells, switching macrophage polarization from M1 to anti-inflammatory M2 phenotype, and inhibiting neutrophils (Li et al., 2005; Tan J.L. et al., 2018). In addition, several studies in animal models have shown that they can exert immunomodulatory effects by inducing other immunosupressive cells, in particular Tregs and Bregs (Manuelpillai et al., 2010; Liu et al., 2012; Tan et al., 2014, 2015; Evans et al., 2018; Li et al., 2018; Tan B. et al., 2018). Moreover, *in vitro* studies showed similar inhibitory effects of hAECs on human cells. For instance, hAECs suppressed human CD4 + T cell proliferation, induced a Th2 cytokine profile, suppressed production of Th1 and Th17 cytokines and promoted differentiation of naïve CD4 + T cells into Tregs (Wolbank et al., 2007; Motedayyen et al., 2018).

Human amniotic epithelial cells have been reported to inhibit immunity by secreting various immunosuppresssive mediators. For example, they produce prostaglandin E_2_ (PGE_2_), which has several immunosuppressive properties such as inhibition of T cell proliferation. hAECs also secrete TGFb, a T cell growth inhibitor and a powerful immunosuppressive molecule (Liu et al., 2012). In addition, the suppressive activity of hAECs has been demonstrated through an increased secretion of the anti-inflammatory cytokine IL-10 (Charles-Henri and Ekaterine, 2020). IL-10 inhibits proinflammatory cytokine production, as well as Th1 and macrophage activation (Howes et al., 2014).

#### Anti-infection and Anti-cancer Properties

Unlike the current therapies for crescentic GN, hAECs have anti-infection properties and protect against cancer development. Human β-defensins, small proteins which promote microbial death, are primarily expressed by epithelial and immune cells at mucosal surfaces (Dorin et al., 2015). These natural anti-microbial molecules have been reported to be secreted by human placenta cells including hAECs and by the fetal membrane during human pregnancy in order to protect the uterus from infection (King et al., 2007; Nemr et al., 2017). hAECs also produce type I interferons (IFNs) in response to viruses *in vitro* (Uchide et al., 2002; Nemr et al., 2017). This group of proteins, which is made up mainly of IFNα and IFNβ, initiate intracellular anti-microbial systems and influence innate and adaptive immune responses (Ivashkiv and Donlin, 2014). They are secreted by infected cells and are important for host protection against viruses through the induction of anti-viral effector molecules (Uchide and Toyoda, 2007). Moreover, hAECs supress cancers directly by inducing apoptosis and reducing motility of malignant cells (Niknejad et al., 2014). They also suppress tumor development indirectly by promoting anti-tumor cytotoxic T cell immunity *in vivo*, as shown in a mouse model of colon adenocarcinoma (Tabatabaei et al., 2018).

#### Protection From Cardiovascular Disease

In contrast to the current drugs used to treat crescentic GN, hAECs also protect against cardiovascular disease. They have decreased areas of myocardial infarction in athymic nude rats (Fang et al., 2012). In addition, administration of hAECs has reduced brain injury in a murine and non-human primate model of ischemic stroke (Evans et al., 2018).

### Human Amniotic Epithelial Cells in Preclinical Studies

#### Human Amniotic Epithelial Cells in Autoimmune Diseases

Administration of hAECs has ameliorated immune-mediated organ damage in models of several autoimmune diseases. hAEC infusion in experimental autoimmune thyroiditis (EAT) and SLE maintained organ function, minimized inflammation and modified the immune balance (Tan B. et al., 2018). In EAT, hAECs reduced disease severity by inhibiting infiltration of inflammatory cells in thyroid glands, as well as suppressing Th17 responses. Moreover, hAECs improved the local cytokine environment in both EAT and SLE mice, by suppressing the levels of IFN-γ and enhancing TGF-β (Tan B. et al., 2018). In SLE mice specifically, hAEC administration promoted Tregs and decreased the levels of pathogenic autoantibodies (Tan B. et al., 2018).

Human amniotic epithelial cell also potently attenuated disease severity in experimental autoimmune encephalomyelitis (EAE), and a mouse model of multiple sclerosis (MS) (Liu et al., 2012). T cell and macrophage infiltration were significantly reduced by hAEC treatment. It was reported that hAECs utilized PGE2 and TGF-β for their immunosuppressive effects (Liu et al., 2012). In another relapsing model of MS, hAECs significantly ameliorated disease progression, while promoting Tregs and augmenting Th2 responses (McDonald et al., 2015).

In an autoimmune uveitis (EAU) rat model, hAECs treatment ameliorated the pathological progression of disease and maintained the retinal structural organization (Li et al., 2018). Infiltration of macrophages and T cells was suppressed after hAEC administration. The stem cells regulated the balance of T cell subsets by decreasing Th17 cells and boosting IL-10-producing Tregs in the spleen and lymph nodes. Furthermore, hAEC treatment changed the ocular chemokine and cytokine environment in EAU rats, indicated by decreased levels of monocyte chemoattractant protein-1, IL-17 and IFN-γ levels, and enhancement of IL-10 (Li et al., 2018).

The immunomodulatory effect of hAECs has also been investigated in mice with autoimmune ovarian disease (AOD) (Zhang Q. et al., 2019). The outcomes showed that hAEC injection improved ovarian function. This was associated with a significant increase in the number of Tregs in the spleen of AOD mice (Zhang Q. et al., 2019).

#### Human Amniotic Epithelial Cells in Other Inflammatory Diseases

Transplantation of both hAECs and their soluble factors have shown beneficial effects in animal models of hepatic fibrosis. hAECs given to mice with induced liver fibrosis reduced hepatocyte apoptosis and decreased hepatic inflammation and fibrosis (Manuelpillai et al., 2010). This study showed that intact cells expressing human-specific markers, inner mitochondrial membrane protein and HLA-G were found in mouse liver 2 weeks following hAEC injection, without evidence of host rejection of the transplanted cells (Manuelpillai et al., 2010). In another study using the same model, hAECs significantly decreased liver fibrosis, in line with reduced hepatic levels of the pro-fibrogenic cytokine TGF-β1, increased expression of the anti-inflammatory mediator IL-10 and decreased hepatic T cell infiltration (Manuelpillai et al., 2012). Furthermore, hAECs administration reduced hepatic macrophage numbers and induced an anti-inflammatory M2 macrophage phenotype (Manuelpillai et al., 2012).

In a rat model of ischemic stroke, hAECs were administered by intracerebral injection, after which they reduced the infarct volume and cerebral apoptosis (Liu et al., 2008). A further study in mice found that hAECs injected 1.5 h after stroke migrated to the ischemic brain and spleen, and limited functional deficit, infarct volume and brain inflammation (Evans et al., 2018). In a rat model of intracerebral hemorrhage, Liang et al. reported that hAECs reduced the levels of proinflammatory cytokines TNF-α and IL-1β in microglia culture medium (Liang et al., 2014). Similarly, in preterm fetal sheep models of brain injury, inflammation was reduced in fetuses that received hAECs (Yawno et al., 2013).

Many studies have explored the immunomodulatory effect of hAECs on lung fibrosis. A study using the bleomycin-induced model of pulmonary fibrosis showed that hAECs can modulate the host inflammatory response, decrease fibrosis and preserve lung function (Murphy et al., 2011). hAECs reduced expression of the pro-inflammatory cytokines TNF-α, IFN-γ and IL-6, and reduced inflammatory cell infiltration (Murphy et al., 2011). A further study using the same model demonstrated that hAEC administration significantly decreased macrophage recruitment into the lung and promoted the majority of alveolar macrophages toward the M2 phenotype (Tan et al., 2014). Moreover, hAECs treatment increased Treg numbers in the injured lungs. It was found that hAECs require Tregs to polarize macrophages toward an M2 phenotype and that hAECs promote Treg cells via TGF-β (Tan et al., 2015).

A recent study in a mouse model of renal ischemia-reperfusion injury, which leads to acute kidney injury, has shown that systematically administered hAECs effectively regulated the kidney immune response (Ren et al., 2020). hAEC infusion attenuated tubular cell death and endothelial necrosis and increased cell proliferation in the injured kidney. The stem cells reprogramed macrophages to shift from a pro-inflammatory M1 to anti-inflammatory M2 phenotype. In addition, hAECs enhanced levels of IL-4 and IL-13 and decreased levels of TNFα and IFNγ, which in turn helped to minimize the inflammatory response.

### Secretome Derived From Human Amniotic Epithelial Cells

The secreted factors (also named secretome) exist in the medium where the stem cells are cultured. Numerous studies on stem cell-derived secreted factors showed that these mediators alone, without the stem cell itself, affect the maturation, migration, polarization and function of immune cells, and thus influence the strength and duration of immune responses. The hAEC secretome contains metabolites, lipids, free nucleic acids, cytokines, growth factors and extracellular matrix proteins. These are all known to play crucial roles in cell-cell communication, acting proximally as well as systemically.

The application of cell-free therapy confers some advantages over stem-cell based applications. Usage of soluble factors bypasses a number of safety concerns potentially linked to the administration of living cell populations including post-transfer mal-transformation, embolism, and transmission of infections. In addition, hAEC secretome may be evaluated for safety, dosage and potency in an approach similar to that used for traditional medications (Vizoso et al., 2017). hAEC-conditioned media (CM) can be also manufactured, packaged, and transported more easily than hAECs themselves (Kay et al., 2017). Therefore, stem cell-derived secretomes have a promising prospect to be used as pharmaceuticals for immune diseases. hAEC CM has shown beneficial effects in reducing a range of conditions by modulating immune responses including liver fibrosis, AOD, inflammatory bowel disease and diabetic wound healing (Hodge et al., 2014; Kuk et al., 2018; Zheng et al., 2018; Zhang Q. et al., 2019). In general, to prepare hAEC CM, hAECs are cultured in chemically defined, serum-free ultraculture medium for 4 days at 37°C in a humidified chamber containing 5% CO_2_, after which conditioned media is harvested and secretome obtained by serial centrifugation (Alhomrani et al., 2017).

Human amniotic epithelial cells also mediate their effects by secreting exosomes. Exosomes are nano-sized biovesicles secreted by various cell types including stem cells under both normal and pathophysiological conditions. They are characterized by a diameter of 50–100 nm and a density of 1.09–1.18 g/mL (Zhang Y. et al., 2019). Exosome cargo is diverse and a pool of exosomes can demonstrate all cellular elements including protein, nucleic acids and lipids (Spada, 2020). They represent a novel manner of intercellular communication, which may play a central role in many cellular processes such as the immune response, signal transduction and antigen presentation (Zhang Y. et al., 2019). Exosomes derived from hAECs produce potent immunomodulatory, anti-fibrotic and pro-regenerative effects and have been successfully used as a cell-free therapy in inflammatory conditions (Alhomrani et al., 2017; Tan J.L. et al., 2018). hAEC-derived exosomes can suppress many immune cells such as T cells, macrophages, and neutrophils *in vitro*. In addition, they have been highly effective in delivering protection from organ damage in a range of disease models including pulmonary and liver fibrosis, as well as acute kidney injury (Alhomrani et al., 2017; Tan J.L. et al., 2018; Ren et al., 2020). Thus, hAEC-derived soluble factors and exosomes may represent a potential cell-free therapy in crescentic GN.

### Human Amniotic Epithelial Cells in Clinical Trials

Based on their immunomodulatory properties, the amniotic membrane and hAECs have been safely used for several years as a therapy for wounds and ocular injuries (Parmar et al., 2006; Jirsova and Jones, 2017). hAECs have also entered clinical trials as a treatment for several conditions including liver fibrosis, stroke and lung injury in premature babies (Table 1; Lim et al., 2017, 2018; Phan et al., 2018; Malhotra et al., 2020). So far, these trials have shown that hAECs are very safe and well-tolerated in humans.

**TABLE 1:** Completed and active clinical trials utilizing hAECs to treat immune-related diseases*.

**Category**	**Registration number**	**Disease**	**Phase**	**Age**	**Country**
Neurology	ACTRN12618000076279	Ischemic stroke	1	18–85 years	Australia
Ophthalmology	NCT00344708	Corneal epithelial dystrophy	N/A	18–88 years	United States
Pneumology	ACTRN12614000174684	Bronchopulmonary dysplasia	1	36 weeks	Australia
Pneumology	ACTRN12618000920291	Bronchopulmonary dysplasia, extremely preterm birth	1	14–18 days	Australia
Orthopedics	NCT03031509	Non-union fracture	1	18–80 years	China
Gynecology	NCT03207412	Premature ovarian failure	N/A	18–40 years	China
Others	ACTRN12616000437460	Cirrhosis, liver fibrosis	1	18–70 years	Australia
Others	ACTRN12618001883202	Crohn’s disease, perianal fistulas	N/A	18–80 years	Australia

*These data were collected from the clinical trial database (ClinicalTrials.gov and anzctr.org.au).*

### Human Amniotic Epithelial Cells as a Potential Therapy for Crescentic Glomerulonephritis

Overall, the studies described above show that hAECs can inhibit organ damage in various autoimmune and inflammatory diseases, without producing major side effects. They exert their effects by inhibiting various pathogenic immune cells including neutrophils, macrophages, effector T cells and B cells, as well as by promoting inhibitory immune subsets such as Tregs and Th2 cells. All these types of immune cells are also involved in the pathogenesis of crescentic GN, thus hAECs may inhibit glomerular injury through similar mechanisms. A summary of the pathways through which hAECs could potentially attenuate crescentic GN is given in Figure 1B.

## Conclusion

In summary, there is a requirement for alternative, safer treatments for crescentic GN as the only effective therapies currently available are broadly immunosuppressive drugs which cause many serious side effects (mainly infections, cancer and cardiovascular problems) and patient deaths. hAECs, as well as hAEC-CM and hAEC-derived exosomes, exert a protective effect in models of various immune-driven conditions, with minimal side effects. They mediate their effects through multiple immunomodulatory and anti-inflammatory mechanisms, and their safety has been proven in clinical trials. Thus, hAEC-based therapy offers promise to be a safe, feasible and effective treatment for crescentic GN. This is due to their (i) ethical and speedy isolation from the placenta which results in an abundance of readily available cells, (ii) unique and selective immunosuppressive capacity, (iii) low immunogenicity, and (iv) ability to fight microbes and cancer and protect against cardiovascular conditions. If proven to be effective in pre-clinical models of crescentic GN, hAECs have the potential to change clinical practice in this disease and provide immense advantages to patients by alleviating their risk of death and complications from unwanted symptoms caused by the existing treatments.

## Author Contributions

AA, JO, and DO wrote the article and agreed with the final version of the manuscript. All authors contributed to the article and approved the submitted version.

## Conflict of Interest

The authors declare that the research was conducted in the absence of any commercial or financial relationships that could be construed as a potential conflict of interest.

## Publisher’s Note

All claims expressed in this article are solely those of the authors and do not necessarily represent those of their affiliated organizations, or those of the publisher, the editors and the reviewers. Any product that may be evaluated in this article, or claim that may be made by its manufacturer, is not guaranteed or endorsed by the publisher.

## References

[B1] Alhomrani M. Correia J. Zavou M. Leaw B. Kuk N. Xu R. (2017). The human amnion epithelial cell secretome decreases hepatic fibrosis in mice with chronic liver fibrosis. *Front. Pharmacol.* 8:748. 10.3389/fphar.2017.00748 29114223 PMC5660722

[B2] Allison A. C. Eugui E. M. (2000). Mycophenolate mofetil and its mechanisms of action. *Immunopharmacology* 47 85–118. 10878285 10.1016/s0162-3109(00)00188-0

[B3] Anders H. J. Saxena R. Zhao M. H. Parodis I. Salmon J. E. Mohan C. (2020). Lupus nephritis. *Nat. Rev. Dis. Primers* 6:7. 31974366 10.1038/s41572-019-0141-9

[B4] Banas R. A. Trumpower C. Bentlejewski C. Marshall V. Sing G. Zeevi A. (2008). Immunogenicity and immunomodulatory effects of amnion-derived multipotent progenitor cells. *Hum. Immunol.* 69 321–328. 10.1016/j.humimm.2008.04.007 18571002

[B5] Ben-David U. Benvenisty N. (2011). The tumorigenicity of human embryonic and induced pluripotent stem cells. *Nat. Rev. Cancer* 11 268–277. 21390058 10.1038/nrc3034

[B6] Bolton W. K. Innes D. J. Jr. Sturgill B. C. Kaiser D. L. (1987). T-cells and macrophages in rapidly progressive glomerulonephritis: clinicopathologic correlations. *Kidney Int.* 32 869–876. 3501499 10.1038/ki.1987.288

[B7] Booth A. Harper L. Hammad T. Bacon P. Griffith M. Levy J. (2004). Prospective study of TNFα blockade with infliximab in anti-neutrophil cytoplasmic antibody-associated systemic vasculitis. *J. Am. Soc. Nephrol.* 15 717–721. 10.1097/01.asn.0000114554.67106.28 14978174

[B8] Caldas H. C. Lojudice F. H. Dias C. Fernandes-Charpiot I. M. M. Baptista M. Kawasaki-Oyama R. S. (2017). Induced pluripotent stem cells reduce progression of experimental chronic kidney disease but develop wilms’. *Tumors. Stem Cells Int.* 2017:7428316. 10.1155/2017/7428316 28845162 PMC5560097

[B9] Chadban S. J. Atkins R. C. (2005). Glomerulonephritis. *Lancet* 365 1797–1806. 15910953 10.1016/S0140-6736(05)66583-X

[B10] Charles-Henri W. Ekaterine B. (2020). Immunomodulatory properties of amniotic membrane derivatives and their potential in regenerative medicine. *Curr. Diab. Rep.* 20:31. 32519069 10.1007/s11892-020-01316-w PMC7283202

[B11] Couser W. G. (1998). Pathogenesis of glomerular damage in glomerulonephritis. *Nephrol. Dial. Transpl.* 13 (Suppl._1), 10–15. 10.1093/ndt/13.suppl_1.10 9507491

[B12] Cunningham M. A. Huang X. R. Dowling J. P. Tipping P. G. Holdsworth S. R. (1999). Prominence of cell-mediated immunity effectors in “pauci-immune” glomerulonephritis. *J. Am. Soc. Nephrol.* 10 499–506. 10.1681/ASN.V103499 10073600

[B13] Daniel M. Peek G. W. Tollefsbol T. O. (2012). Regulation of the human catalytic subunit of telomerase (hTERT). *Gene* 498 135–146. 22381618 10.1016/j.gene.2012.01.095 PMC3312932

[B14] Dean E. G. Wilson G. R. Li M. Edgtton K. L. O’Sullivan K. Hudson B. G. (2005). Experimental autoimmune Goodpasture’s disease: a pathogenetic role for both effector cells and antibody in injury. *Kidney Int.* 67 566–575. 10.1111/j.1523-1755.2005.67113.x 15673304

[B15] Dey B. Dange P. Ganesh R. N. Parameswaran S. Sivan Pillai P. P. (2016). Immune-complex deposits in anti-neutrophil cytoplasmic antibody associated crescentic glomerulonephritis; a report of two cases. *Immunopathol. Persa* 3:e09.

[B16] Disteldorf E. M. Krebs C. F. Paust H. J. Turner J. E. Nouailles G. Tittel A. (2015). CXCL5 drives neutrophil recruitment in TH17-mediated GN. *J. Am. Soc. Nephrol.* 26 55–66. 10.1681/ASN.2013101061 24904089 PMC4279732

[B17] Dorin J. R. McHugh B. J. Cox S. L. Davidson D. J. (2015). Mammalian antimicrobial peptides; defensins and cathelicidins. *Mol. Med. Microbiol.* 1 539–565.

[B18] D’Souza M. J. Oettinger C. W. Shah A. Tipping P. G. Huang X. R. Milton G. V. (1999). Macrophage depletion by albumin microencapsulated clodronate: attenuation of cytokine release in macrophage-dependent glomerulonephritis. *Drug Dev. Ind. Pharm.* 25 591–596. 10.1081/ddc-100102213 10219527

[B19] Evans M. A. Lim R. Kim H. A. Chu H. X. Gardiner-Mann C. V. Taylor K. W. (2018). Acute or delayed systemic administration of human amnion epithelial cells improves outcomes in experimental stroke. *Stroke* 49 700–709. 29382802 10.1161/STROKEAHA.117.019136

[B20] Falk R. Jennette J. (1988). Anti-neutrophil cytoplasmic autoantibodies with specificity for myeloperoxidase in patients with systemic vasculitis and idiopathic necrotizing and crescentic glomerulonephritis. *N. Engl. J. Med.* 318 1651–1657. 2453802 10.1056/NEJM198806233182504

[B21] Fang C.-H. Jin J. Joe J.-H. Song Y.-S. So B.-I. Lim S. M. (2012). In vivo differentiation of human amniotic epithelial cells into cardiomyocyte-like cells and cell transplantation effect on myocardial infarction in rats: comparison with cord blood and adipose tissue-derived mesenchymal stem cells. *Cell Transplant.* 21 1687–1696. 10.3727/096368912X653039 22776022

[B22] Flossmann O. Berden A. de Groot K. Hagen C. Harper L. Heijl C. (2011). Long-term patient survival in ANCA-associated vasculitis. *Ann. Rheum. Dis.* 70 488–494. 21109517 10.1136/ard.2010.137778

[B23] Furuhashi K. Tsuboi N. Shimizu A. Katsuno T. Kim H. Saka Y. (2013). Serum-starved adipose-derived stromal cells ameliorate crescentic GN by promoting immunoregulatory macrophages. *J. Am. Soc. Nephrol.* 24 587–603. 10.1681/ASN.2012030264 23471196 PMC3609131

[B24] Gan P. Y. Steinmetz O. M. Tan D. S. O’Sullivan K. M. Ooi J. D. Iwakura Y. (2010). Th17 cells promote autoimmune anti-myeloperoxidase glomerulonephritis. *J. Am. Soc. Nephrol.* 21 925–931. 20299361 10.1681/ASN.2009070763 PMC2900960

[B25] Geng X. D. Zheng W. Wu C. M. Wang S. Q. Hong Q. Cai G. Y. (2016). Embryonic stem cells-loaded gelatin microcryogels slow progression of chronic kidney disease. *Chin. Med. J.* 129 392–398. 10.4103/0366-6999.176088 26879011 PMC4800838

[B26] Gulati K. McAdoo S. P. (2018). Anti-glomerular basement membrane disease. *Rheum Dis. Clin. North Am.* 44 651–673. 30274629 10.1016/j.rdc.2018.06.011

[B27] Hamour S. Gan P. Y. Pepper R. Florez Barros F. Wang H. H. O’Sullivan K. (2015). Local IL-17 production exerts a protective role in murine experimental glomerulonephritis. *PLoS One* 10:e0136238. 10.1371/journal.pone.0136238 26317864 PMC4552867

[B28] Herrnstadt G. Steinmetz O. (2020). The role of Treg subtypes in glomerulonephritis. *Cell Tissue Res.* 10.1007/s00441-020-03359-7 Online ahead of print 33315130 PMC8523467

[B29] Hodge A. Lourensz D. Vaghjiani V. Nguyen H. Tchongue J. Wang B. (2014). Soluble factors derived from human amniotic epithelial cells suppress collagen production in human hepatic stellate cells. *Cytotherapy* 16 1132–1144. 10.1016/j.jcyt.2014.01.005 24642017

[B30] Holdsworth S. R. Neale T. J. Wilson C. B. (1981). Abrogation of macrophage-dependent injury in experimental glomerulonephritis in the rabbit: use of an antimacrophage serum. *J. Clin. Invest.* 68 686–698. 10.1172/jci110304 7276168 PMC370850

[B31] Hori J. Wang M. Kamiya K. Takahashi H. Sakuragawa N. (2006). Immunological characteristics of amniotic epithelium. *Cornea* 25 S53–S58. 17001194 10.1097/01.ico.0000247214.31757.5c

[B32] Howes A. Stimpson P. Redford P. Gabrysova L. O’Garra A. (2014). “Interleukin-10: cytokines in anti-inflammation and tolerance,” in *Cytokine Frontiers*, eds Yoshimoto T. Yoshimoto T. (Tokyo: Springer), 327–352.

[B33] Hu W. Liu Z. Chen H. Tang Z. Wang Q. Shen K. (2002). Mycophenolate mofetil vs cyclophosphamide therapy for patients with diffuse proliferative lupus nephritis. *Chin. Med. J.* 115 705–709. 12133539

[B34] Huang X. Tipping P. G. Apostolopoulos J. Oettinger C. D’souza M. Milton G. (1997). Mechanisms of T cell-induced glomerular injury in anti-glomeruler basement membrane (GBM) glomerulonephritis in rats. *Clin. Exp. Immunol.* 109 134–142. 9218836 10.1046/j.1365-2249.1997.4091307.x PMC1904710

[B35] Huang X.-R. Tipping P. G. Shuo L. Holdsworth S. R. (1997). Th1 responsiveness to nephritogenic antigens determines susceptibility to crescentic glomerulonephritis in mice. *Kidney Int.* 51 94–103. 8995722 10.1038/ki.1997.12

[B36] Humrich J. Y. Morbach H. Undeutsch R. Enghard P. Rosenberger S. Weigert O. (2010). Homeostatic imbalance of regulatory and effector T cells due to IL-2 deprivation amplifies murine lupus. *Proc. Natl. Acad. Sci. U.S.A.* 107 204–209. 10.1073/pnas.0903158107 20018660 PMC2806746

[B37] Imig J. D. Ryan M. J. (2013). Immune and inflammatory role in renal disease. *Compr. Physiol.* 3 957–976. 23720336 10.1002/cphy.c120028 PMC3803162

[B38] Ivashkiv L. B. Donlin L. T. (2014). Regulation of type I interferon responses. *Nat. Rev. Immunol.* 14 36–49. 24362405 10.1038/nri3581 PMC4084561

[B39] Jefferson J. A. (2018). Complications of immunosuppression in glomerular disease. *Clin. J. Am. Soc. Nephrol.* 13 1264–1275. 30042223 10.2215/CJN.01920218 PMC6086710

[B40] Jennette J. C. (2003). Rapidly progressive crescentic glomerulonephritis. *Kidney Int.* 63 1164–1177. 12631105 10.1046/j.1523-1755.2003.00843.x

[B41] Jennette J. C. Thomas D. (2001). Crescentic glomerulonephritis. *Nephrol. Dial. Transplant.* 16 (Suppl._6), 80–82. 11568252 10.1093/ndt/16.suppl_6.80

[B42] Jhaveri K. D. Shah H. H. Calderon K. Campenot E. S. Radhakrishnan J. (2013). Glomerular diseases seen with cancer and chemotherapy: a narrative review. *Kidney Int.* 84 34–44. 10.1038/ki.2012.484 23364518

[B43] Jiang W. Xu J. (2020). Immune modulation by mesenchymal stem cells. *Cell Prolif.* 53:e12712. 31730279 10.1111/cpr.12712 PMC6985662

[B44] Jin M. Xie Y. Li Q. Chen X. (2014). Stem cell-based cell therapy for glomerulonephritis. *BioMed. Res. Int.* 2014:124730. 25003105 10.1155/2014/124730 PMC4070530

[B45] Jirsova K. Jones G. L. (2017). Amniotic membrane in ophthalmology: properties, preparation, storage and indications for grafting—a review. *Cell Tissue Bank.* 18 193–204. 10.1007/s10561-017-9618-5 28255771

[B46] Johnson R. Couser W. G. Alpers C. E. Vissers M. Schulze M. Klebanoff S. J. (1988). The human neutrophil serine proteinases, elastase and cathepsin G, can mediate glomerular injury in vivo. *J. Exp. Med.* 168 1169–1174. 10.1084/jem.168.3.1169 3049904 PMC2189047

[B47] Jones R. B. Furuta S. Tervaert J. W. Hauser T. Luqmani R. Morgan M. D. (2015). Rituximab versus cyclophosphamide in ANCA-associated renal vasculitis: 2-year results of a randomised trial. *Ann. Rheum. Dis.* 74 1178–1182. 10.1136/annrheumdis-2014-206404 25739829

[B48] Kallenberg C. G. (2014). Key advances in the clinical approach to ANCA-associated vasculitis. *Nat. Rev. Rheumatol.* 10 484–493. 24980139 10.1038/nrrheum.2014.104

[B49] Kaplan M. J. (2013). Role of neutrophils in systemic autoimmune diseases. *Arthritis Res. Ther.* 15:219. 24286137 10.1186/ar4325 PMC3978765

[B50] Karras A. (2018). Microscopic polyangiitis: new insights into pathogenesis, clinical features and therapy. *Semin. Respir. Crit. Care Med.* 39 459–464. 30404112 10.1055/s-0038-1673387

[B51] Kay A. G. Long G. Tyler G. Stefan A. Broadfoot S. J. Piccinini A. M. (2017). Mesenchymal stem cell-conditioned medium reduces disease severity and immune responses in inflammatory arthritis. *Sci. Rep.* 7:18019. 10.1038/s41598-017-18144-w 29269885 PMC5740178

[B52] King A. Paltoo A. Kelly R. Sallenave J.-M. Bocking A. Challis J. (2007). Expression of natural antimicrobials by human placenta and fetal membranes. *Placenta* 28 161–169. 16513165 10.1016/j.placenta.2006.01.006

[B53] King C. Harper L. (2017). Avoidance of harm from treatment for ANCA-associated vasculitis. *Curr. Treatm. Opt. Rheumatol.* 3 230–243. 10.1007/s40674-017-0082-y 29201630 PMC5694500

[B54] Kitching A. R. Holdsworth S. R. Tipping P. G. (1999). IFN-γ mediates crescent formation and cell-mediated immune injury in murine glomerulonephritis. *J. Am. Soc. Nephrol.* 10 752–759. 10.1681/ASN.V104752 10203359

[B55] Kitching A. R. Holdsworth S. R. Tipping P. G. (2000). Crescentic glomerulonephritis-a manifestation of a nephritogenic Thl response? *Histol. Histopathol.* 15 993–1003. 10.14670/HH-15.993 10963141

[B56] Kitching A. R. Tipping P. G. Mutch D. A. Huang X. R. Holdsworth S. R. (1998). Interleukin-4 deficiency enhances Th1 responses and crescentic glomerulonephritis in mice. *Kidney Int.* 53 112–118. 9453006 10.1046/j.1523-1755.1998.00733.x

[B57] Kitching A. R. Turner A. L. Semple T. Li M. Edgtton K. L. Wilson G. R. (2004). Experimental autoimmune anti-glomerular basement membrane glomerulonephritis: a protective role for IFN-gamma. *J. Am. Soc. Nephrol.* 15 1764–1774. 15213264 10.1097/01.asn.0000128968.27705.5e

[B58] Kuk N. Correia J. Alhomrani M. Lim R. Sievert W. Hodge A. (2018). DOP060 Human amnion epithelial cells and their conditioned media reduces intestinal inflammation and fibrosis in a murine model of chronic colitis. *J. Crohns. Colitis* 12 (Suppl._1), S072–S.

[B59] Lan H. Nikolic-Paterson D. Mu W. Atkins R. (1997). Local macrophage proliferation in the pathogenesis of glomerular crescent formation in rat anti-glomerular basement membrane (GBM) glomerulonephritis. *Clin. Exp. Immunol.* 110 233–240. 9367407 10.1111/j.1365-2249.1997.tb08322.x PMC2265489

[B60] Laurino S. Chaudhry A. Booth A. Conte G. Jayne D. (2010). Prospective study of TNFα blockade with adalimumab in ANCA-associated systemic vasculitis with renal involvement. *Nephrol. Dial. Transplant.* 25 3307–3314. 20368305 10.1093/ndt/gfq187

[B61] Lefebvre S. Adrian F. Moreau P. Gourand L. Dausset J. Berrih-Aknin S. (2000). Modulation of HLA-G expression in human thymic and amniotic epithelial cells. *Hum. Immunol.* 61 1095–1101. 10.1016/s0198-8859(00)00192-0 11137212

[B62] Li H. Niederkorn J. Y. Neelam S. Mayhew E. Word R. A. McCulley J. P. (2005). Immunosuppressive factors secreted by human amniotic epithelial cells. *Invest. Ophthalmol. Vis. Sci.* 46 900–907. 15728546 10.1167/iovs.04-0495

[B63] Li J. Qiu C. Zhang Z. Yuan W. Ge Z. Tan B. (2018). Subretinal transplantation of human amniotic epithelial cells in the treatment of autoimmune uveitis in rats. *Cell Transplant.* 27 1504–1514. 10.1177/0963689718796196 30168350 PMC6180726

[B64] Li S. Holdsworth S. R. Tipping P. G. (1997). Antibody independent crescentic glomerulonephritis in μ chain deficient mice. *Kidney Int.* 51 672–678. 10.1038/ki.1997.97 9067898

[B65] Liang H. Guan D. Gao A. Yin Y. Jing M. Yang L. (2014). Human amniotic epithelial stem cells inhibit microglia activation through downregulation of tumor necrosis factor-α, interleukin-1β and matrix metalloproteinase-12 in vitro and in a rat model of intracerebral hemorrhage. *Cytotherapy* 16 523–534. 10.1016/j.jcyt.2013.11.007 24424266

[B66] Lim R. Hodge A. Moore G. Wallace E. M. Sievert W. (2017). A pilot study evaluating the safety of intravenously administered human amnion epithelial cells for the treatment of hepatic fibrosis. *Front. Pharmacol.* 8:549. 10.3389/fphar.2017.00549 28878671 PMC5572339

[B67] Lim R. Malhotra A. Tan J. Chan S. T. Lau S. Zhu D. (2018). First-in-human administration of allogeneic amnion cells in premature infants with bronchopulmonary dysplasia: a safety study. *Stem Cells Transl. Med.* 7 628–635. 10.1002/sctm.18-0079 30078207 PMC6127230

[B68] Liu T. Wu J. Huang Q. Hou Y. Jiang Z. Zang S. (2008). Human amniotic epithelial cells ameliorate behavioral dysfunction and reduce infarct size in the rat middle cerebral artery occlusion model. *Shock* 29 603–611. 10.1097/SHK.0b013e318157e845 18414234

[B69] Liu Y. H. Vaghjiani V. Tee J. Y. To K. Cui P. Oh D. Y. (2012). Amniotic epithelial cells from the human placenta potently suppress a mouse model of multiple sclerosis. *PLoS One* 7:e35758. 10.1371/journal.pone.0035758 22563398 PMC3338525

[B70] Lu G. Zhang X. Shen L. Qiao Q. Li Y. Sun J. (2017). CCL20 secreted from IgA1-stimulated human mesangial cells recruits inflammatory Th17 cells in IgA nephropathy. *PLoS One* 12:e0178352. 10.1371/journal.pone.0178352 28552941 PMC5446182

[B71] Luger D. Silver P. B. Tang J. Cua D. Chen Z. Iwakura Y. (2008). Either a Th17 or a Th1 effector response can drive autoimmunity: conditions of disease induction affect dominant effector category. *J. Exp. Med.* 205 799–810. 18391061 10.1084/jem.20071258 PMC2292220

[B72] Ma X. Che N. Gu Z. Huang J. Wang D. Liang J. (2013). Allogenic mesenchymal stem cell transplantation ameliorates nephritis in lupus mice via inhibition of B-cell activation. *Cell Transplant.* 22 2279–2290. 10.3727/096368912X658692 23127285

[B73] Mahdi B. M. (2013). A glow of HLA typing in organ transplantation. *Clin. Transl. Med.* 2:6. 23432791 10.1186/2001-1326-2-6 PMC3598844

[B74] Malhotra A. Lim R. Mockler J. C. Wallace E. M. (2020). Two-year outcomes of infants enrolled in the first-in-human study of amnion cells for bronchopulmonary dysplasia. *Stem Cells Transl. Med.* 9 289–294. 10.1002/sctm.19-0251 31774236 PMC7031636

[B75] Manuelpillai U. Lourensz D. Vaghjiani V. Tchongue J. Lacey D. Tee J.-Y. (2012). Human amniotic epithelial cell transplantation induces markers of alternative macrophage activation and reduces established hepatic fibrosis. *PLoS One* 7:e38631. 10.1371/journal.pone.0038631 22719909 PMC3375296

[B76] Manuelpillai U. Tchongue J. Lourensz D. Vaghjiani V. Samuel C. S. Liu A. (2010). Transplantation of human amnion epithelial cells reduces hepatic fibrosis in immunocompetent CCl4-treated mice. *Cell Transplant.* 19 1157–1168. 10.3727/096368910X504496 20447339

[B77] McAdoo S. P. Pusey C. D. (2017). Anti-glomerular basement membrane disease. *Clin. J. Am. Soc. Nephrol.* 12 1162–1172. 28515156 10.2215/CJN.01380217 PMC5498345

[B78] McDonald C. A. Payne N. L. Sun G. Moussa L. Siatskas C. Lim R. (2015). Immunosuppressive potential of human amnion epithelial cells in the treatment of experimental autoimmune encephalomyelitis. *J. Neuroinflammation* 12:112. 10.1186/s12974-015-0322-8 26036872 PMC4457975

[B79] Mejia-Vilet J. Parikh S. (2019). “Overview of the current approach to glomerular disease classification,” in *Glomerulonephritis*, eds Trachtman H. Herlitz L. Lerma E. Hogan J. (Cham: Springer), 59–85.

[B80] Ménétrier-Caux C. Ray-Coquard I. Blay J. Y. Caux C. (2019). Lymphopenia in cancer patients and its effects on response to immunotherapy: an opportunity for combination with cytokines? *J. Immunother. Cancer.* 7:85. 10.1186/s40425-019-0549-5 30922400 PMC6437964

[B81] Menez S. P. El Essawy B. Atta M. G. (2018). Lupus nephritis: current treatment paradigm and unmet needs. *Rev. Recent. Clin. Trials.* 13 105–113. 29173182 10.2174/1574887112666171123113200

[B82] Miki T. Lehmann T. Cai H. Stolz D. B. Strom S. C. (2005). Stem cell characteristics of amniotic epithelial cells. *Stem Cells* 23 1549–1559. 16081662 10.1634/stemcells.2004-0357

[B83] Moroni G. Ponticelli C. (2014). Rapidly progressive crescentic glomerulonephritis: early treatment is a must. *Autoimmun. Rev.* 13 723–729. 24657897 10.1016/j.autrev.2014.02.007

[B84] Motedayyen H. Zarnani A. H. Tajik N. Ghotloo S. Rezaei A. (2018). Immunomodulatory effects of human amniotic epithelial cells on naive CD4(+) T cells from women with unexplained recurrent spontaneous abortion. *Placenta* 71 31–40. 30415745 10.1016/j.placenta.2018.06.008

[B85] Müller G. A. Müller C. A. Markovic-Lipkovski J. Kilper R. B. Risler T. (1988). Renal, major histocompatibility complex antigens and cellular components in rapidly progressive glomerulonephritis identified by monoclonal antibodies. *Nephron* 49 132–139. 10.1159/000185039 3288888

[B86] Murphy S. Lim R. Dickinson H. Acharya R. Rosli S. Jenkin G. (2011). Human amnion epithelial cells prevent bleomycin-induced lung injury and preserve lung function. *Cell Transplant.* 20 909–924. 10.3727/096368910X543385 21092408

[B87] Murray I. R. Péault B. (2015). Q&A: mesenchymal stem cells - where do they come from and is it important? *BMC Biol.* 13:99. 26596888 10.1186/s12915-015-0212-7 PMC4656175

[B88] Mûzes G. Sipos F. (2019). Issues and opportunities of stem cell therapy in autoimmune diseases. *World J. Stem Cells.* 11 212–221. 31110602 10.4252/wjsc.v11.i4.212 PMC6503459

[B89] Naik R. H. Shawar S. H. (2020). *Rapidly Progressive Glomerulonephritis.* Treasure Island, FL: StatPearls Publishing. 32491362

[B90] Nemr W. Bashandy M. Araby E. Khamiss O. (2017). Molecular displaying of differential immunoresponse to various infections of amniotic epithelia. *Am. J. Reprod. Immunol.* 77:e12662. 10.1111/aji.12662 28378902

[B91] Niknejad H. Khayat-Khoei M. Peirovi H. Abolghasemi H. (2014). Human amniotic epithelial cells induce apoptosis of cancer cells: a new anti-tumor therapeutic strategy. *Cytotherapy* 16 33–40. 10.1016/j.jcyt.2013.07.005 24113429

[B92] Nolasco E. F. Cameron J. S. Hartley B. Coelho A. Hildreth G. Reuben R. (1987). Intraglomerular T cells and monocytes in nephritis: study with monoclonal antibodies. *Kidney Int.* 31 1160–1166. 3496476 10.1038/ki.1987.123

[B93] Nomura S. Sasaki T. Kitano Y. Osawa G. Niederstadt C. Lerche L. (1996). The critical role of intercellular adhesion molecule-1 in masugi nephritis in rats. *Nephron* 73 264–272. 10.1159/000189050 8773354

[B94] Odobasic D. Gan P. Y. Summers S. A. Semple T. J. Muljadi R. C. Iwakura Y. (2011). Interleukin-17A promotes early but attenuates established disease in crescentic glomerulonephritis in mice. *Am. J. Pathol.* 179 1188–1198. 10.1016/j.ajpath.2011.05.039 21741931 PMC3157183

[B95] Odobasic D. Ghali J. R. O’Sullivan K. M. Holdsworth S. R. Kitching A. R. (2014). Glomerulonephritis induced by heterologous anti-GBM globulin as a planted foreign antigen. *Curr. Protoc. Immunol.* 106 15.26.1–15.26.20. 10.1002/0471142735.im1526s106 25081909

[B96] Odobasic D. Kitching A. R. Semple T. J. Holdsworth S. R. (2007). Endogenous myeloperoxidase promotes neutrophil-mediated renal injury, but attenuates T cell immunity inducing crescentic glomerulonephritis. *J. Am. Soc. Nephrol.* 18 760–770. 10.1681/ASN.2006040375 17267745

[B97] Odobasic D. Oudin V. Ito K. Gan P. Y. Kitching A. R. Holdsworth S. R. (2019). Tolerogenic dendritic cells attenuate experimental autoimmune antimyeloperoxidase glomerulonephritis. *J. Am. Soc. Nephrol.* 30 2140–2157. 10.1681/ASN.2019030236 31444274 PMC6830784

[B98] Olnes M. J. Kotliarov Y. Biancotto A. Cheung F. Chen J. Shi R. (2016). Effects of systemically administered hydrocortisone on the human immunome. *Sci. Rep.* 6:23002. 26972611 10.1038/srep23002 PMC4789739

[B99] Ooi J. D. Gan P. Y. Chen T. Eggenhuizen P. J. Chang J. Alikhan M. A. (2014). FcgammaRIIB regulates T-cell autoreactivity, ANCA production, and neutrophil activation to suppress anti-myeloperoxidase glomerulonephritis. *Kidney Int.* 86 1140–1149. 10.1038/ki.2014.189 24869670

[B100] Ooi J. D. Phoon R. K. Holdsworth S. R. Kitching A. R. (2009). IL-23, not IL-12, directs autoimmunity to the Goodpasture antigen. *J. Am. Soc. Nephrol.* 20 980–989. 19357249 10.1681/ASN.2008080891 PMC2678043

[B101] Ooi J. D. Snelgrove S. L. Engel D. R. Hochheiser K. Ludwig-Portugall I. Nozaki Y. (2011). Endogenous foxp3+ T-regulatory cells suppress anti-glomerular basement membrane nephritis. *Kidney Int.* 79 977–986. 10.1038/ki.2010.541 21248715

[B102] Ostmann A. Paust H. J. Panzer U. Wegscheid C. Kapffer S. Huber S. (2013). Regulatory T cell-derived IL-10 ameliorates crescentic GN. *J. Am. Soc. Nephrol.* 24 930–942. 10.1681/ASN.2012070684 23641052 PMC3665390

[B103] Ougaard M. K. E. Kvist P. H. Jensen H. E. Hess C. Rune I. Søndergaard H. (2018). Murine nephrotoxic nephritis as a model of chronic kidney disease. *Int. J. Nephrol.* 2018:8424502. 29692933 10.1155/2018/8424502 PMC5859794

[B104] Parmar D. N. Alizadeh H. Awwad S. T. Li H. Neelam S. Bowman R. W. (2006). Ocular surface restoration using non-surgical transplantation of tissue-cultured human amniotic epithelial cells. *Am. J. Ophthalmol.* 141 299.–307. 10.1016/j.ajo.2005.09.008 16458684

[B105] Parmar M. S. Bashir K. (2017). *Crescentric Glomerulonephritis.* Treasure Island, FL: StatPearls Publishing. 28613478

[B106] Parmar M. S. Bashir K. (2019). *Crescentric Glomerulonephritis.* Treasure Island, FL: StatPearls Publishing. 28613478

[B107] Paust H.-J. Turner J.-E. Steinmetz O. M. Peters A. Heymann F. Hölscher C. (2009). The IL-23/Th17 axis contributes to renal injury in experimental glomerulonephritis. *J. Am. Soc. Nephrol.* 20 969–979. 19339380 10.1681/ASN.2008050556 PMC2678032

[B108] Phan T. G. Ma H. Lim R. Sobey C. G. Wallace E. M. (2018). Phase 1 trial of amnion cell therapy for ischemic stroke. *Front. Neurol.* 9:198. 10.3389/fneur.2018.00198 29930530 PMC5999782

[B109] Ponticelli C. Locatelli F. (2018). Glucocorticoids in the treatment of glomerular diseases: pitfalls and pearls. *Clin. J. Am. Soc. Nephrol.* 13 815–822. 10.2215/CJN.12991117 29475991 PMC5969489

[B110] Prendecki M. Pusey C. (2019). Plasma exchange in anti-glomerular basement membrane disease. *Presse Med.* 48 328–337. 10.1016/j.lpm.2019.03.017 31703956

[B111] Qiu C. Ge Z. Cui W. Yu L. Li J. (2020). Human amniotic epithelial stem cells: a promising seed cell for clinical applications. *Int. J. Mol. Sci.* 21:7730. 10.3390/ijms21207730 33086620 PMC7594030

[B112] Ren Y. Chen Y. Zheng X. Wang H. Kang X. Tang J. (2020). Human amniotic epithelial cells ameliorate kidney damage in ischemia-reperfusion mouse model of acute kidney injury. *Stem Cell Res. Therapy* 11 410. 10.1186/s13287-020-01917-y 32967729 PMC7510147

[B113] Richard M. L. Gilkeson G. (2018). Mouse models of lupus: what they tell us and what they don’t. *Lupus Sci. Med.* 5:e000199. 10.1136/lupus-2016-000199 29387435 PMC5786947

[B114] Richards H. B. Satoh M. Jennette J. C. Croker B. P. Yoshida H. Reeves W. H. (2001). Interferon-gamma is required for lupus nephritis in mice treated with the hydrocarbon oil pristane. *Kidney Int.* 60 2173–2180. 10.1046/j.1523-1755.2001.00045.x 11737591

[B115] Rousselle A. Kettritz R. Schreiber A. (2017). Monocytes promote crescent formation in anti-myeloperoxidase antibody-induced glomerulonephritis. *Am. J. Pathol.* 187 1908–1915. 28667835 10.1016/j.ajpath.2017.05.003

[B116] Ruth A. Kitching A. Kwan R. Odobasic D. Ooi J. Timoshanko J. (2006). Anti-neutrophil cytoplasmic antibodies and effector CD4+ cells play nonredundant roles in anti-myeloperoxidase crescentic glomerulonephritis. *J. Am. Soc. Nephrol.* 17 1940–1949. 10.1681/ASN.2006020108 16769746

[B117] Ryu J. S. Jeong E. J. Kim J. Y. Park S. J. Ju W. S. Kim C. H. (2020). Application of mesenchymal stem cells in inflammatory and fibrotic diseases. *Int. J. Mol. Sci.* 21:8366. 10.3390/ijms21218366 PMC7664655 33171878

[B118] Saleem S. Dai Z. Coelho S. N. Konieczny B. T. Assmann K. J. Baddoura F. K. (1998). IL-4 is an endogenous inhibitor of neutrophil influx and subsequent pathology in acute antibody-mediated inflammation. *J. Immunol.* 160 979–984. 9551937

[B119] Santos J. E. Fiel D. Santos R. Vicente R. Aguiar R. Santos I. (2020). Rituximab use in adult glomerulopathies and its rationale. *J. Bras. Nephrol.* 42 77–93. 10.1590/2175-8239-JBN-2018-0254 31904761 PMC7213927

[B120] Schrijver G. Bogman M. J. J. Assmann K. J. de Waal R. M. Robben H. C. van Gasteren H. (1990). Anti-GBM nephritis in the mouse: role of granulocytes in the heterologous phase. *Kidney Inter.* 38 86–95. 10.1038/ki.1990.171 2385089

[B121] Sheu J. J. Sung P. H. Wallace C. G. Yang C. C. Chen K. H. Shao P. L. (2020). Intravenous administration of iPS-MSC(SPIONs) mobilized into CKD parenchyma and effectively preserved residual renal function in CKD rat. *J. Cell Mol. Med.* 24 3593–3610. 10.1111/jcmm.15050 32061051 PMC7131913

[B122] Spada S. (2020). Methods to purify DNA from extracellular vesicles: focus on exosomes. *Methods Enzymol.* 645 109–118. 10.1016/bs.mie.2020.09.004 33565966

[B123] Stachura I. Si L. Whiteside T. L. (1984). Mononuclear-cell subsets in human idiopathic crescentic glomerulonephritis (ICGN): analysis in tissue sections with monoclonal antibodies. *J. Clin. Immunol.* 4 202–208. 10.1007/BF00914967 6610688

[B124] Summers S. A. Steinmetz O. M. Gan P. Y. Ooi J. D. Odobasic D. Kitching A. R. (2011). Toll-like receptor 2 induces Th17 myeloperoxidase autoimmunity while Toll-like receptor 9 drives Th1 autoimmunity in murine vasculitis. *Arthritis Rheum.* 63 1124–1135. 10.1002/art.30208 21190299

[B125] Summers S. A. Steinmetz O. M. Li M. Kausman J. Y. Semple T. Edgtton K. L. (2009). Th1 and Th17 cells induce proliferative glomerulonephritis. *J. Am. Soc. Nephrol.* 20 2518–2524. 19820122 10.1681/ASN.2009030337 PMC2794236

[B126] Suzuki T. Iyoda M. Shibata T. Ohtaki H. Matsumoto K. Shindo-Hirai Y. (2013). Therapeutic effects of human mesenchymal stem cells in Wistar-Kyoto rats with anti-glomerular basement membrane glomerulonephritis. *PLoS One* 8:e67475. 10.1371/journal.pone.0067475 23826305 PMC3691173

[B127] Syed R. Rehman A. Valecha G. El-Sayegh S. (2015). Pauci-immune crescentic glomerulonephritis: an ANCA-associated vasculitis. *BioMed Res. Int.* 2015:402826. 26688808 10.1155/2015/402826 PMC4673333

[B128] Tabatabaei M. Mosaffa N. Ghods R. Nikoo S. Kazemnejad S. Khanmohammadi M. (2018). Vaccination with human amniotic epithelial cells confer effective protection in a murine model of colon adenocarcinoma. *Int. J. Cancer* 142 1453–1466. 10.1002/ijc.31159 29139122

[B129] Tan B. Yuan W. Li J. Yang P. Ge Z. Liu J. (2018). Therapeutic effect of human amniotic epithelial cells in murine models of Hashimoto’s thyroiditis and Systemic lupus erythematosus. *Cytotherapy* 20 1247–1258. 10.1016/j.jcyt.2018.04.001 30174233

[B130] Tan D. S. Gan P. Y. O’Sullivan K. M. Hammett M. V. Summers S. A. Ooi J. D. (2013). Thymic deletion and regulatory T cells prevent antimyeloperoxidase GN. *J. Am. Soc. Nephrol.* 24 573–585. 10.1681/ASN.2012090898 23393320 PMC3609139

[B131] Tan J. L. Chan S. T. Lo C. Y. Deane J. A. McDonald C. A. Bernard C. C. (2015). Amnion cell-mediated immune modulation following bleomycin challenge: controlling the regulatory T cell response. *Stem Cell Res. Ther.* 6:8. 10.1186/scrt542 25634246 PMC4417266

[B132] Tan J. L. Chan S. T. Wallace E. M. Lim R. (2014). Human amnion epithelial cells mediate lung repair by directly modulating macrophage recruitment and polarization. *Cell Transplant.* 23 319–328. 10.3727/096368912X661409 23294809

[B133] Tan J. L. Lau S. N. Leaw B. Nguyen H. P. T. Salamonsen L. A. Saad M. I. (2018). Amnion epithelial cell-derived exosomes restrict lung injury and enhance endogenous lung repair. *Stem Cells Transl. Med.* 7 180–196. 10.1002/sctm.17-0185 29297621 PMC5788876

[B134] Thakkar U. G. Vanikar A. V. Trivedi H. L. (2017). Stem cell therapy: an emerging modality in glomerular diseases. *Cytotherapy* 19 333–348. 10.1016/j.jcyt.2016.11.003 28089754

[B135] Tipping P. G. Huang X. R. Berndt M. C. Holdsworth S. R. (1994). A role for P selectin in complement-independent neutrophil-mediated glomerular injury. *Kidney Inter.* 46 79–88. 10.1038/ki.1994.246 7523757

[B136] Tipping P. G. Huang X. R. Qi M. Van G. Y. Tang W. W. (1998). Crescentic glomerulonephritis in CD4-and CD8-deficient mice. Requirement for CD4 but not CD8 cells. *Am. J. Pathol.* 152:1541. 9626058 PMC1858447

[B137] Tögel F. Hu Z. Weiss K. Isaac J. Lange C. Westenfelder C. (2005). Administered mesenchymal stem cells protect against ischemic acute renal failure through differentiation-independent mechanisms. *Am. J. Physiol. Renal. Physiol.* 289 F31–F42. 10.1152/ajprenal.00007.2005 15713913

[B138] Tsui C. Dokouhaki P. Prasad B. (2018). Fibrillary glomerulonephritis with crescentic and necrotizing glomerulonephritis and concurrent thrombotic microangiopathy. *Case Rep. Nephrol. Dial.* 8 182–191. 10.1159/000492529 30320122 PMC6167697

[B139] Tumlin J. A. Lohavichan V. Hennigar R. (2003). Crescentic, proliferative IgA nephropathy: clinical and histological response to methylprednisolone and intravenous cyclophosphamide. *Nephrol. Dial. Transplant.* 18 1321–1329. 12808169 10.1093/ndt/gfg081

[B140] Uchide N. Ohyama K. Yuan B. Sano T. Bessho T. Yamakawa T. (2002). Differential mRNA expression of inflammatory cytokines in cultured human fetal membrane cells responding to influenza virus infection. *Biol. Pharm. Bull.* 25 239–243. 10.1248/bpb.25.239 11853174

[B141] Uchide N. Toyoda H. (2007). Current status of monocyte differentiation-inducing (MDI) factors derived from human fetal membrane chorion cells undergoing apoptosis after influenza virus infection. *Gene Regul. Syst. Biol.* 1 295–302. 10.4137/grsb.s374 19936095 PMC2759142

[B142] Vinen C. Oliveira D. (2003). Acute glomerulonephritis. *Postgrad. Med. J.* 79 206–213. 12743337 10.1136/pmj.79.930.206 PMC1742671

[B143] Vizoso F. J. Eiro N. Cid S. Schneider J. Perez-Fernandez R. (2017). Mesenchymal stem cell secretome: toward cell-free therapeutic strategies in regenerative medicine. *Int. J. Mol. Sci.* 18:1852. 10.3390/ijms18091852 PMC5618501 28841158

[B144] Wang Y. Chen X. Cao W. Shi Y. (2014). Plasticity of mesenchymal stem cells in immunomodulation: pathological and therapeutic implications. *Nat. Immunol.* 15 1009–1016. 25329189 10.1038/ni.3002

[B145] Wegener’s Granulomatosis Etanercept Trial (WGET) Research Group (2005). Etanercept plus standard therapy for Wegener’s granulomatosis. *N. Engl. J. Med.* 352 351–361. 10.1056/NEJMoa041884 15673801

[B146] Wolbank S. Peterbauer A. Fahrner M. Hennerbichler S. van Griensven M. Stadler G. (2007). Dose-dependent immunomodulatory effect of human stem cells from amniotic membrane: a comparison with human mesenchymal stem cells from adipose tissue. *Tissue Eng.* 13 1173–1183. 10.1089/ten.2006.0313 17518752

[B147] Xiao H. Heeringa P. Hu P. Liu Z. Zhao M. Aratani Y. (2002). Antineutrophil cytoplasmic autoantibodies specific for myeloperoxidase cause glomerulonephritis and vasculitis in mice. *J. Clin. Invest.* 110 955–963. 12370273 10.1172/JCI15918 PMC151154

[B148] Yang C. Huang X.-R. Fung E. Liu H.-F. Lan H.-Y. (2017). The regulatory T-cell transcription factor Foxp3 protects against crescentic glomerulonephritis. *Sci. Rep.* 7:1481. 10.1038/s41598-017-01515-8 28469165 PMC5431186

[B149] Yawno T. Schuilwerve J. Moss T. J. Vosdoganes P. Westover A. J. Afandi E. (2013). Human amnion epithelial cells reduce fetal brain injury in response to intrauterine inflammation. *Dev. Neurosci.* 35 272–282. 23571644 10.1159/000346683

[B150] Zhang Q. Huang Y. Sun J. Gu T. Shao X. Lai D. (2019). Immunomodulatory effect of human amniotic epithelial cells on restoration of ovarian function in mice with autoimmune ovarian disease. *Acta Biochim. Biophys. Sin.* 51 845–855. 10.1093/abbs/gmz065 31287492

[B151] Zhang Y. Liu Y. Liu H. Tang W. (2019). Exosomes: biogenesis, biologic function and clinical potential. *Cell Biosci.* 9:19. 30815248 10.1186/s13578-019-0282-2 PMC6377728

[B152] Zheng Y. Zheng S. Fan X. Li L. Xiao Y. Luo P. (2018). Amniotic epithelial cells accelerate diabetic wound healing by modulating inflammation and promoting neovascularization. *Stem Cells Int.* 2018:1082076. 10.1155/2018/1082076 30210547 PMC6120261

